# How the Mid-Victorians Worked, Ate and Died†

**DOI:** 10.3390/ijerph6031235

**Published:** 2009-03-20

**Authors:** Paul Clayton, Judith Rowbotham

**Affiliations:** 1 School of Life Sciences, Oxford Brookes University, UK; 2 Department of History & Law, Nottingham Trent University, UK; E-Mail: jrowbotham@gmail.com

**Keywords:** Public health, dietary shift, degenerative disease, Victorian

## Abstract

Analysis of the mid-Victorian period in the U.K. reveals that life expectancy at age 5 was as good or better than exists today, and the incidence of degenerative disease was 10% of ours. Their levels of physical activity and hence calorific intakes were approximately twice ours. They had relatively little access to alcohol and tobacco; and due to their correspondingly high intake of fruits, whole grains, oily fish and vegetables, they consumed levels of micro- and phytonutrients at approximately ten times the levels considered normal today. This paper relates the nutritional status of the mid-Victorians to their freedom from degenerative disease; and extrapolates recommendations for the cost-effective improvement of public health today.

## 1. Introduction

The mid-Victorian period is usually defined as the years between 1850 and 1870, but in nutritional terms we have identified a slightly longer period, lasting until around 1880. During these 30 years, we argue here, a generation grew up with probably the best standards of health ever enjoyed by a modern state. The British population had risen significantly and had become increasingly urbanised, but the great public health movement had not yet been established and Britain’s towns and cities were still notoriously unhealthy environments [4,5]. Despite this, and contrary to historical tradition, we argue in this paper, using a range of historical evidence, which Britain and its world-dominating empire were supported by a workforce, an army and a navy comprised of individuals who were healthier, fitter and stronger than we are today. They were almost entirely free of the degenerative diseases which maim and kill so many of us, and although it is commonly stated that this is because they all died young, the reverse is true; public records reveal that they lived as long – or longer – than we do in the 21^st^ century.

These findings are remarkable, as this brief period of great good health predates not only the public health movement but also the great 20^th^ century medical advances in surgery, infection control and drugs [6–8]. They are also in marked contrast to popular views about Victorian squalor and disease, views that have long obscured the realities of life and death during that ‘period of equipoise’ [9].

Our recent research indicates that the mid-Victorians’ good health was entirely due to their superior diet. This period was, nutritionally speaking, an island in time; one that was created and subsequently squandered by economic and political forces. This begs a series of questions. How did this brief nutritional ‘golden age’ come about? How was it lost? And could we recreate it?

One key contributory factor was what used to be called the Agricultural Revolution; a series of developments in agricultural practice that massively improved crop and livestock yields. This slow green revolution started in the late seventeenth century, gradually accelerated into the mid-19^th^ century, and underpinned both modern urbanisation and the associated Industrial Revolution [10]. Arguably the most critical agricultural development was a more complex system of crop rotation, which greatly improved both arable output and animal husbandry. In the 1730’s a new breed of innovative land-owner (epitomised by Marquis ‘Turnip’ Townshend) introduced new systems of crop rotation from Sweden and The Netherlands, and new crops like the swede (*Brassica napus napobrassica*). The new crop rotation systems avoided the need to let land lie fallow one year in three, and instead used a four or five year cycle in which turnips and clover were used as two of the crops because of their ability to replenish the soil. These new systems created immense gains in food productivity. Between 1705 and 1765 English wheat exports increased ten-fold, while the increased availability of animal feed meant that most livestock no longer had to be slaughtered at the onset of winter so that fresh (instead of salted) meat became cheaper and more widely available throughout the year [11].

Population shifts also played a key contributory role. The bulk of the population had always lived on the land but by 1850, as revealed by the 1851 census, more Britons were living and working in towns than in the countryside [4]. The agricultural improvements of the previous 150 years meant that agriculture produced far more than before, but used far fewer people to achieve this. As a result, people moved to towns to find work: Britain was the first modern consumer society and there was real demand for workers in an increasing number of urban industries [12]. Traditionally, urban life expectancy was significantly lower than rural life expectancy, but from the mid-Victorian period on this difference disappears.

Victorian society was very different to traditional society. It was a class society as we understand it today rather than the older, more deferential model, and this created enormous social tensions though it is important not to exaggerate these [13]. For the very poor, towns remained deeply unpleasant places to live, and it can be argued that for many, the social structure of towns even got worse. As more of the working classes moved into towns, more of the middle classes moved out to create the beginnings of suburbia [14]. The great Victorian commentator Thomas Carlyle claimed that in cities, little tied one human being to another except for the ‘Cash Nexus’, where employer and employee met in an uncomfortable wage and profit-driven relationship [15], as Mrs Gaskell revealed in books like *North and South* [16].

In many ways, however, urban socio-economic conditions were getting better by the mid-century. Trades unions and philanthropists were slowly but surely improving urban working conditions and wages throughout the last half of the century [17]. The threats of political instability which had seemed most threatening in towns up to the late 1840s were largely dispersed during the mid-Victorian era, as a result of changes in the political and legal systems. For example, the Great Reform Act of 1832 was followed by the 1867 Reform Act, which meant that most male urban heads of households were now able to vote. In 1845 the notorious Corn Laws were finally repealed ushering in the era of cheap food for the urban masses.

One of the most important results of these changes was that the interests of the landed classes were no longer protected. Traditionally, parliament had always sought to protect the income of farmers and landowners, and after the end of the Napoleonic Wars, this stance had seen the introduction of the highly unpopular Corn Laws from 1815. These kept the price of grain at a level that ensured agricultural prosperity, but they had a disastrous effect on the price of food. This particularly affected the new urban, industrial workforce, which was heavily dependent on bread as a staple food. The Corn Laws kept the price of bread artificially high, even during economic depressions such as the 1840s, a decade which became notorious as the ‘Hungry 40’s’ [18].

The post-Great Reform Act parliament, however, was susceptible to pressure from groups such as the Anti-Corn Law League led by Richard Cobden and Joseph Bright. When the situation was exacerbated by the Irish Great Potato Famine, Prime Minister Sir Robert Peel, the grandson of a mill-owner, forced through the repeal of the Corn Laws [18]. From that time on farming interests were under pressure to produce cheap food because it had become clear that the prosperity of the country depended on industrial rather than on agricultural output [19]. As the Great Exhibition of 1851 underlined, Britain had become the Workshop of the World [20].

Improved agricultural output and a political climate dedicated to ensuring cheap food led to a dramatic increase in the production of affordable foodstuffs; but it was the development of the railway network that actually brought the fruits of the agricultural and political changes into the towns and cities, and made them available to the mid-Victorian working classes [21].

The start of the modern railway age is usually marked by the opening of the Stockton & Darlington line in 1825. From the late 1830s on, progress was impressively rapid. Important long-distance lines came first, followed by smaller local lines criss-crossing the country. The London and Birmingham line opened in 1838, part of Brunel's London to Bristol route the same year and the London and Southampton line in 1840. By the mid century the key lines were already laid. The railway system grew exponentially, reaching 2500 miles by 1845, and continued to expand, carrying goods as well as passengers. Thanks to trains, producers were now supplying the urban markets with more, fresher and cheaper food than was previously possible. This boosted urban demand for fresh foodstuffs, and pushed up agricultural output still further [22]. A survey of food availability in the 1860s through sources such as Henry Mayhew’s survey of the London poor shows very substantial quantities of affordable vegetables and fruits now pouring into the urban markets [23].

This fortunate combination of factors produced a sea change in the nation, and in the nation’s health. By 1850 Britain’s increasing domestic productivity and foreign power had created a national mood of confidence and optimism which affected all levels of society. Driven by better nutrition, far more than the new schemes of clean air and water which were only beginning to have an effect from the 1870s on, adult life expectancy increased from the 1850s until by 1875 it matched or surpassed our own [24]. The health and vitality of the British population during this period was reflected in the workforces and armed forces that powered the transformation of the urban landscape at home, and drove the great expansion of the British Empire abroad [20].

Unfortunately, negative changes that would undermine these nutritional gains were already taking shape. Thanks to her dominant global position, and developments in shipping technology, Britain had created a global market drawing in the products of colonial and US agriculture, to provide ever-cheaper food for the growing urban masses. From 1875 on and especially after 1885, rising imports of cheap food basics were increasingly affecting the food chain at home. Imported North American wheat and new milling techniques reduced the prices of white flour and bread. Tinned meat arrived from the Argentine, Australia and New Zealand, which was cheaper than either home-produced or refrigerated fresh meat also arriving from these sources. Canned fruit and condensed milk became widely available [25].

This expansion in the range of foods was advertised by most contemporaries, and by subsequent historians, as representing a significant ‘improvement’ in the working class diet. The reality was very different. These changes undoubtedly increased the variety and quantity of the working class diet, but its quality deteriorated markedly. The imported canned meats were fatty and usually *‘*corned’ or salted. Cheaper sugar promoted a huge increase in sugar consumption in confectionery, now mass-produced for the first time, and in the new processed foods such as sugar-laden condensed milk, and canned fruits bathed in heavy syrup. The increased sugar consumption caused such damage to the nation’s teeth that by 1900 it was commonly noted that people could no longer chew tough foods and were unable to eat many vegetables, fruits and nuts [26]. For all these reasons the late-Victorian diet actually damaged the health of the nation, and the health of the working classes in particular.

The decline was astonishingly rapid. The mid-Victorian navvies, who as seasonal workers were towards the bottom end of the economic scale, could routinely shovel up to 20 tons of earth per day from below their feet to above their heads [27]. This was an enormous physical effort that required great strength, stamina and robust good health. Within two generations, however, male health nationally had deteriorated to such an extent that in 1900, five out of 10 young men volunteering for the second Boer War had to be rejected because they were so undernourished. They were not starved, but had been consuming the wrong foods [28,29]. This reality is underlined by considering army recruitment earlier. The recruiting sergeants had reported no such problems during previous high profile campaigns such as the Asante (1873–4) and Zulu (1877–8) Wars [30].

The fall in nutritional standards between 1880 and 1900 was so marked that the generations were visibly and progressively shrinking. In 1883 the infantry were forced to lower the minimum height for recruits from 5ft 6 inches to 5ft 3 inches. This was because most new recruits were now coming from an urban background instead of the traditional rural background (the 1881 census showed that over three-quarters of the population now lived in towns and cities). Factors such as a lack of sunlight in urban slums (which led to rickets due to Vitamin D deficiency) had already reduced the height of young male volunteers. Lack of sunlight, however, could not have been the sole critical factor in the next height reduction, a mere 18 years later. By this time, clean air legislation had markedly improved urban sunlight levels; but unfortunately, the supposed ‘improvements’ in dietary intake resulting from imported foods had had time to take effect on the 16–18 year old cohort. It might be expected that the infantry would be able to raise the minimum height requirement back to 5ft. 6 inches. Instead, they were forced to reduce it still further, to a mere 5ft. British officers, who were from the middle and upper classes and not yet exposed to more than the occasional treats of canned produce, were far better fed in terms of their intake of fresh foods and were now on average a full head taller than their malnourished and sickly men.

In 1904, and as a direct result of the Boer disaster, the government set up the Committee on Physical Deterioration. Its report, emphasising the need to provide school meals for working class children, reinforced the idea that the urban working classes were not only malnourished at the start of the twentieth century but also (in an unjustified leap of the imagination, reinforced by folk memories of the ‘Hungry 40’s) that they had been so since the start of nineteenth century industrial urbanisation [28,31]. This profound error of thought was incorporated into subsequent models of public health, and is distorting and damaging healthcare to this day.

The crude average figures often used to depict the brevity of Victorian lives mislead because they include infant mortality, which was tragically high. If we strip out peri-natal mortality, however, and look at the life expectancy of those who survived the first five years, a very different picture emerges. Victorian contemporary sources reveal that life expectancy for adults in the mid-Victorian period was almost exactly what it is today. At 65, men could expect another ten years of life; and women another eight [24,32,33] (the lower figure for women reflects the high danger of death in childbirth, mainly from causes unrelated to malnutrition). This compares surprisingly favourably with today’s figures: life expectancy at birth (reflecting our improved standards of neo-natal care) averages 75.9 years (men) and 81.3 years (women); though recent work has suggested that for working class men and women this is lower, at around 72 for men and 76 for women [34].

If we accept the working class figures, which are probably more directly comparable with the Victorian data, women have gained three years of life expectancy since the mid-Victorian period while men have actually fallen back by 3 years. The decline in male life expectancy implicates several causal factors; including the introduction of industrialised cigarette production in 1883, a sustained fall in the relative cost of alcohol and a severe decline in nutritional standards, as outlined below. The improvement in female life expectancy can be partly linked to family planning developments but also to other factors promoting women’s health such as improvements in dress. Until widespread accessible family planning facilities arrived after the First World War, women’s health could be substantially undermined by up to 30 years of successive pregnancies and births [35–37]. These figures suggest that if twentieth century women had not also experienced the negative impacts of tobacco consumption becoming respectable, along with an increased alcohol intake and worsening nutrition as they began to consume the imported delicacies originally preserved mainly for the men (all those things which had cost their menfolk three years), they would have gained six years.

Given that modern pharmaceutical, surgical, anaesthetic, scanning and other diagnostic technologies were self-evidently unavailable to the mid-Victorians, their high life expectancy is very striking, and can only have been due to their health-promoting lifestyle. But the implications of this new understanding of the mid-Victorian period are rather more profound. It shows that medical advances allied to the pharmaceutical industry’s output have done little more than change the manner of our dying. The Victorians died rapidly of infection and/or trauma, whereas we die slowly of degenerative disease. It reveals that with the exception of family planning, the vast edifice of twentieth century healthcare has not enabled us to live longer but has in the main merely supplied methods of suppressing the symptoms of degenerative diseases which have emerged due to our failure to maintain mid-Victorian nutritional standards [38]. Above all, it refutes the Panglossian optimism of the contemporary anti-ageing movement whose protagonists use 1900 – a nadir in health and life expectancy trends - as their starting point to promote the idea of endlessly increasing life span. These are the equivalent of the get-rich-quick share pushers who insisted, during the dot.com boom, that we had at last escaped the constraints of normal economics. Some believed their own message of eternal growth; others used it to sell junk bonds they knew were worthless. The parallels with today’s vitamin pill market are obvious, but this also echoes the way in which Big Pharma trumpets the arrival of each new miracle drug.

In short, the majority of even the poorest mid-Victorians lived well, despite all their disadvantages and what we would now consider discomforts. Those that survived the perils of childbirth and infancy lived as long as we do, and were healthier while they were alive their prolonged good health was due to their high levels of physical activity, and as a consequence, how and what they ate. We could learn a good deal from them.

## 2. How the Mid-Victorians Worked

Due to the high levels of physical activity routinely undertaken by the Victorian working classes, calorific requirements ranged between 150 and 200% of today’s historically low values. Almost all work involved moderate to heavy physical labour, and often included that involved in getting to work. Seasonal and other low-paid workers often had to walk up to six miles per day [39]. While some Victorian working class women worked from home (seamstressing for instance) more went out to work in shops, factories and workshops, necessitating long days on their feet, plus the additional burden of housework [39,40]. Many single women were domestics, either live-in servants or daily workers. This was particularly physically demanding, as very few households had male servants, so women did all the heavy household work from scrubbing floors to heaving coals upstairs. Men worked on average 9–10 hours/day, for 5.5 to 6 days a week, giving a range from 50 to 60 hours of physical activity per week [40]. Factoring in the walk to and from work increases the range of total hours of work-related physical activity up to 55 to 70 hours per week. Women’s expenditure of effort was similarly large [41]. Married women had also domestic chores in their own homes after work, and in addition, their daily dress up to the 1890s at least (when the development of the tailor-made costume reduced both corseting and the weight of numerous layers of fabric) involved real physical effort just in moving around. Male leisure activities such as gardening and informal football also involved substantial physical effort.

Using average figures for work-related calorie consumption, men required between 280 (walking) and 440 calories (heavy yard work) per hour; with women requiring between 260 and 350 calories per hour. This gives calorific expenditure ranges during the working week of between 3,000 to 4,500 calories /day (men) and 2,750 to 3,500 (women).

Total calorific requirements were likely to have been even higher during the winter months; with less insulated and less warmed homes, working class Victorians used more calories to keep warm than we do. The same held true for workplaces, unless the work (certain factory operations, blacksmithing, etc) heated the environment to unhealthy levels. At the top end of the physical activity range were the ‘navigators’, the labourers who built (largely without machinery) the roads and railways that enabled the expansion of the British economy. These men were expending 5,000 calories or more per day.

In short, the mid-Victorians ate twice as much as we do, but due to their high levels of physical activity remained slim; overweight and obesity were relatively rare, and (unless associated with ill-health) were generally identified as phenomenona associated with the numerically smaller middle and upper-middle class. But it is not just the amount of food the mid-Victorians consumed that is so unfamiliar; the composition of their diet was also very different from our own.

## 3. What the Mid-Victorians Ate

### 

#### Vegetables, Green and Root

Onions were amongst the cheapest vegetables, widely available all year around at a cost so negligible that few housewives budgeted what cost them around a halfpenny (even cheaper if bruised) for a bunch containing at least a dozen. They might become slightly more expensive in the late spring, when leeks could be substituted [41]. Watercress was another cheap staple in the working class diet, available at a halfpenny for four bunches in the period April to February [41]. The Jerusalem artichoke was consumed from September through to March, often home-grown as it was one of the easiest vegetables to grow in urban allotments [42]. Carrots and turnips were inexpensive staples, especially during the winter months. Cabbage was also cheap and readily available, along with broccoli. Fresh peas were available and affordable from June to July, with beans from July to September [41].

#### Fruit

Apples were the cheapest and most commonly available urban fruits from August through to May; with cherries taking over in the May– July period, followed by gooseberries in June, up to August, then plums and greengages in July through to September [41]. Dried fruits and candied peel were always cheaply available, and used to sweeten desserts such as bread puddings and for cakes and mincemeat. They were also consumed as an afternoon snack, particularly by children, according to Victorian cookery books [42,43] and many other sources from Dickens to Mayhew. All fruits and vegetables were organically grown, and therefore had higher levels of phytonutrients than the intensively grown crops we eat today [44].

#### Legumes and Nuts

Dried legumes were available all year round, and widely used (e.g. pease pudding). The chestnut was the most commonly consumed nut and one of the most commonly eaten street snacks in the chestnut season, running from September through to January. Filberts or hazelnuts were available from October through to May; walnuts were another regularly bought seasonal nut. Imported almonds and Brazil nuts were more expensive, but widely consumed around Christmas as a ‘treat’. Coconuts were also imported, often given as presents or won at fairs; commonly grated for use in cakes and desserts [23].

#### Fish and Seafoods

The herring was one of the most important fish in the Victorian urban diet; fresh in the autumn, winter and spring; dried and salted (red herring) or pickled/soused all year round. Red herrings were a staple of the working class diet throughout the year because they were easily cooked (e.g. Idylls of the Poor). Other favourites were cheap and easily obtainable varieties with better keeping qualities than the more vulnerable white fish, including sprats, eels, and shellfish (oysters, mussels, cockles, whelks). Of the white fish consumed, cod, haddock and John Dory were preferred. Typically, and unlike today, the whole fish was consumed including heads and roes [22]. Fish was available from Monday evening to Friday evening; with broken and day old fish or eels and shoreline shellfish available on Saturdays, as fishermen did not go out over the weekends [45].

#### Meats

Consumption of meat was considered a mark of a good diet and its complete absence was rare: consuming only limited amounts was a poverty diet [23]. Joints of meat were, for the poor, likely to be an occasional treat. Yet only those with the least secure incomes and most limited housing, and so without either the cooking facilities or the funds, would be unlikely to have a weekly Sunday joint; even they might achieve that three or four times a year, cooked in a local cookhouse or bakery oven. Otherwise, meat on the bone (shin or cheek), stewed or fried, was the most economical form of meat, generally eked out with offal meats including brains, heart, sweetbreads, liver, kidneys and ‘pluck’, (the lungs and intestines of sheep). Pork was the most commonly consumed meat. All meats were from free-range animals.

#### Eggs and Dairy Products

Many East End households kept hens in their backyards, and Robert’s study of Lancashire suggests similar patterns [36]. Keeping a couple of hens could produce up to a dozen eggs per household per week. There were fears about adulteration of milk (frequently watered-down). Butter did not feature largely in the working-class diet. Dripping was a preferred substitute in the days before cheap margarine. Hard cheeses, as opposed to soft cheeses, were favoured by the working classes as a regular part of their diet, partly because even when the heel of the cheese was too hard to eat, the ends could be toasted.

#### Alcohol

Beer was the most commonly consumed form of alcohol, but with an alcohol content significantly lower than today’s beers. Careful reading of contemporary sources including cookery and domestic economy books suggest that the alcohol percent of beer consumed in the home was probably only 1% to 2%; often less as it was watered down, especially for consumption by women and children [43,46,47]. In pubs, the alcohol content of beer was more regulated and generally higher, ranging from 2% to 3%. These are still weak beers, compared to today’s average of around 5%. Spirits were more intermittently consumed by men and rarely by women: respectability and gin did not go together [48]. Working class men and women seldom drank wine, except for port or sherry. A third or more of households were temperate or teetotal, partly due to the sustained efforts of the anti-alcohol movement. [49,50].

#### Tobacco

Pipe smoking was widespread but intermittent amongst working class males, and a cigar or cheroot might be smoked on special occasions. Snuff had largely fallen out of favour, as had chewing tobacco. The big expansion in mass tobacco consumption by the working classes did not take place until after 1883, when industrial cigarette production was introduced [51]. It was not until the twentieth century that women of all classes became major consumers of tobacco, under the pressure of heavy advertising.

#### Adulterants

Some adulterants commonly used in Victorian foods were well-known to be toxic even then: lead chromate in mustard, mercury and arsenic compounds as colourants in confectionery and picrotoxin in beer all undoubtedly contributed to ill health. In contrast, modern nutritional biochemistry reveals that some of the other common ‘adulterants’ have potentially significant health benefits. The hawthorne used to extend tea, for example, contained vaso- and cardio-protective flavonoids [52–57]. The coriander in beer may have had some anthelmintic activity [58], and the watering down of beer and spirits was – from a health perspective – a generally good thing!

#### Dietary Summary

Mid-Victorian working class men and women consumed between 50% and 100% more calories than we do, but because they were so much more physically active than we are today, overweight and obesity hardly existed at the working class level. The working class diet was rich in seasonal vegetables and fruits; with consumption of fruits and vegetables amounting to eight to 10 portions per day. This far exceeds the current national average of around three portions, and the government-recommended five-a-day. The mid-Victorian diet also contained significantly more nuts, legumes, whole grains and omega three fatty acids than the modern diet. Much meat consumed was offal, which has a higher micronutrient density than the skeletal muscle we largely eat today [59]. Prior to the introduction of margarine in the late Victorian period, dietary intakes of trans fats were very low. There were very few processed foods and therefore little hidden salt, other than in bread (Recipes suggest that significantly less salt was then added to meals. At table, salt was not usually sprinkled on a serving but piled at the side of the plate, allowing consumers to regulate consumption in a more controlled way.). The mid-Victorian diet had a lower calorific density and a higher nutrient density than ours. It had a higher content of fibre (including fermentable fibre), and a lower sodium/potassium ratio. In short, the mid-Victorians ate a diet that was not only considerably better than our own, but also far in advance of current government recommendations. It more closely resembles the Mediterranean diet, proven in many studies to promote health and longevity; or even the ‘Paleolithic diet’ recommended by some nutritionists [60].

In terms of alcohol consumption, the comparisons with today are also revealing. Many contemporary reports suggest that around a fifth of Victorian working class men might, when employed, spend up to a fifth of their income on beer [61]. Assuming an average urban income ranging from £1 to £4 per week, and given mid-century pub prices of 3d to 8d per pint for beer, the reported expenditure would account for around 16 pints to 20 per week maximum or between three and four pints per night. As Victorian beer generally had an alcohol content ranging between 1 and 3.5% [62], this is equivalent to one and a half to two pints of beer per day in contemporary terms. Seen in this light, the huge Victorian concerns about drunkenness in the Victorian working classes appear to be more a reflection of respectable morality than a real public health issue [63]. Cost implications ensured that for most, the Victorian ‘alcohol problem’ was certainly less significant than it is in our time, when the frequency of public drunkenness and levels of injury and illness have become a serious public health concern (64). Finally, mid-Victorian tobacco consumption was very much lower than today.

These new findings reveal that, contrary to received wisdom, the mid-Victorians ate a healthier diet than we do today. This had dramatic effects on their health and life expectancy.

## 4. How the Mid-Victorians Died

### 

#### Public Health Patterns

The overall pattern of Victorian causes of death broadly resembles that found in developing countries today, with infection, trauma and infant/mother mortality in the pole positions, and non-communicable degenerative disease being relatively insignificant.

Common causes of death [65,66]

1. Infection including TB and other lung infections such as pneumonia; epidemics (scarlet fever, smallpox, influenza, typhoid, cholera etc), with spread often linked to poor sanitation: and the sexually transmitted diseases.
2. Accidents/trauma linked to work place and domestic conditions. Death from burns was an important cause of death among women, due largely to a combination of open hearth cooking, fashions in dress, and the use of highly flammable fabrics.
3. Infant/mother mortality [66]. This was generally due to infection, although maternal haemorrhage was another significant causative factor.
4. Heart failure. This was generally due to damage to the heart valves caused by rheumatic fever, and was not a degenerative disease. Angina pectoris does not appear in the registrar general’s records as a cause of death until 1857 – and then as a disease of old age - although the diagnosis and its causes were recognised [67–70].

Uncommon causes of death

5. Coronary artery disease (see above)
6. Paralytic fits (strokes, see *Webster’s Dictionary*). Stroke was mainly associated with the middle and upper classes who ate a diet in which animal derived foods had a more significant role, and who consumed as a result rather less fruits and vegetables. Strokes were generally non-fatal, at least the first time; although mortality rates increased with each subsequent stroke [65]
7. Cancers were relatively rare [65]. While the Victorians did not possess sophisticated diagnostic or screening technology, they were as able to diagnose late stage cancer as we are today; but this was an uncommon finding. In that period, cancer carried none of the stigma that it has recently acquired, and was diagnosed without bias. For example, in 1869 the Physician to Charing Cross Hospital describes lung cancer as ‘… one of the rarer forms of a rare disease. You may probably pass the rest of your students life without seeing another example of it.’ [71].

Not only were cancers very uncommon compared to today, they appear to have differed in other key respects. James Paget (of Paget’s Disease) built a large practice on the strength of diagnosing breast cancer, which he did by sight and palpation – that is at Stages 3 and 4. In this group he describes a life expectancy of 4 years after diagnosis, extending to eight or more with surgery [72]. The corresponding figures today are Stage 3: 50% survival at 10 years if given surgery, chemo- and radio-therapy, and Stage 4: overall survival about 15 months. These figures suggest that breast cancer during the Victorian period was significantly less rapidly progressive than is the case today, probably due to the Victorians’ significantly higher intakes of a range of micro- and phytonutrients which slow cancer growth.

In summary, although the mid-Victorians lived as long as we do, they were relatively immune to the chronic degenerative diseases that are the most important causes of ill health and death today.

## 5. What Did the Victorians Ever Do for Us?

The implications of the mid-Victorian story are far-reaching, because, unlike the paleolithic scenario, details of the mid-Victorian lifestyle and its impact on public health are extensively documented. Thus, the mid-Victorian experience clearly shows us that:

1. Degenerative diseases are not caused by old age (the ‘wear and tear’ hypothesis); but are driven, in the main, by chronic malnutrition. Our low energy lifestyles leave us depleted in anabolic and anti-catabolic co-factors; and this imbalance is compounded by excessive intakes of inflammatory compounds. The current epidemic of degenerative disease is caused by widespread problem of multiple micro- and phyto-nutrient depletion (Type B malnutrition.)
2. With the exception of family planning and antibiotics, the vast edifice of twentieth century healthcare has generated little more than tools to suppress symptoms of the degenerative diseases which have emerged due to our failure to maintain mid-Victorian nutritional standards.
3. The only way to combat the adverse effects of Type B malnutrition, and to prevent and / or cure degenerative disease, is to enhance the nutrient density of the modern diet.

## 6. The Case for Supplements

Our levels of physical activity and therefore our food intakes are at an historic low. To make matters worse, when compared to the mid-Victorian diet, the modern diet is rich in processed foods. It has a higher sodium/potassium ratio, and contains far less fruit, vegetables, whole grains and omega 3 fatty acids. It is lower in fibre and phytonutrients, in proportional and absolute terms; and, because of our high intakes of potato products, breakfast cereals, confectionery and refined baked goods, may have a higher glycemic load. Given all this, it follows that we are inevitably more likely to suffer from dysnutrition (multiple micro- and phytonutrient depletion) than our mid-Victorian forebears.

This is supported by survey findings on both sides of the Atlantic; the U.S.D.A.’s 1994 to 1996 Continuing Survey of Food Intakes by Individuals [73,74], and the National Diet and Nutrition Surveys [75] both show that many individuals today are unable to obtain RNI values of a variety of vitamins and minerals. Malnutrition in the U.K. is now reckoned to contribute to illness-related costs in excess of £7.3 billion per annum [76]. Since it would be unacceptable and impractical to recreate the mid-Victorian working class 4,000 calorie/day diet, this constitutes a persuasive argument for a more widespread use of food fortification and/or properly designed food supplements (most supplements on the market are incredibly badly designed; they are assembled by companies that do not understand the real nutritional issues that confront us today, and sell us pills containing irrational combinations and doses that can do more harm than good [77]).

To insist, as orthodox nutritionists and dieticians do, that only whole fruit and veg contain the magical, health-promoting ingredients represents little more than the last gasp of the discredited and anti-scientific theory of vitalism (‘Vitalism—the insistence that there is some big, mysterious extra ingredient in all living things—turns out to have been not a deep insight but a failure of imagination’, Daniel Dennett) [78]. Even the stately FSA concedes that fruit juices count towards your five-a-day, as do freeze-dried powdered extracts of fruits and vegetables. As our knowledge of phytochemistry and phytopharmacology increases, it has become perfectly acceptable to use rational combinations of the key plant constituents in pill or capsule form.

These arguments are developed in ‘Pharmageddon’ [79], a medical textbook which illustrates how micro- and phyto-nutrients can be specifically combined in order to prevent and treat the chronic degenerative diseases that characterise and dominate the 20^th^ and 21^st^ centuries; and how they could be integrated into our food chain in order to reduce the contemporary and excessively high risks of the degenerative diseases to the far lower mid-Victorian levels.

## 7. Final Comment

In light of the huge body of evidence linking diet to health, many researchers are now studying the dietary intakes of different groups of people and attempting to tease out such esoteric factors as, for example, just how much omega 3 fish oil is necessary to reduce the risk of Alzheimer’s; or how what dose of flavonoids should be consumed to reduce the risk of stomach cancer.

Most of this research is patently a waste of time. Current generations are, from an historical point of view, anomalous. Our historically low levels of physical activity and consequently food intakes mean that even those groups consuming the highest levels of berry fruits, green leaf vegetables or oily fish, are still well below optimal (mid-Victorian) levels of consumption.

For example, eminent scientists working with dietary elements thought to reduce the risk of cancer have commented that although ‘pharmacological levels’ of compounds such as flavonoids or salicylates have strong anti-cancer properties in vitro, there is little evidence that dietary (or ‘physiological’) levels of intake have any protective effects in humans.

In contrast the mid-Victorians, with their far greater intakes of fruits and vegetables, which were organic and in many cases contained significantly higher concentrations of phytonutrients than our intensively grown crops do [80–85] were consuming ‘pharmacological’ levels of these valuable and protective compounds. This would explain why they were so effectively protected against cancer, and heart disease, and *all* the other degenerative, non-communicable disorders. And it would also explain why, with our very low ‘physiological’ intakes, we are so terribly prone to these largely avoidable diseases.

We believe also that the on-going search for disease susceptibility genes is ahistoric and therefore largely misinformed. The mid-Victorian gene pool was not significantly different to our own, yet their incidence of degenerative disease was approximately 90% less [24]. In the high-nutrient mid-Victorian environment, the vast majority of the population was protected; and the combination of high levels of physical activity and an excellent diet enhanced the expression of a coordinated array of health-promoting genes [86,87]. As the nutrient tide has receded, increasing numbers of genetic polymorphisms have become exposed. [88], making current genome-wide association studies (GWAS) largely redundant (If we take this argument to an extreme, and progress to a diet totally devoid of micronutrients, all polymorphisms become disease-associated.). It follows that the pharmaceutical industry’s attempts to develop genomically derived and individualized treatments such as RNA interference and ISPC are unlikely to impact on public health. The steel vessel of Public Health is rent open, and the drug companies are selling us high-priced pots of caulk.

Do not, therefore, look to the drug companies to provide remedies for the appalling state of our health; nor to our politicians who seem unable, in many cases, to see far beyond the brims of their parliamentary troughs. Look, instead, to the food and beverage industries, and to a lesser extent the supplement companies, who may well step up to the plate with better designed foods and nutritional programmes once the currently profoundly counter-productive regulatory system has been re-drafted.

## Figures and Tables

**Figure 1.**
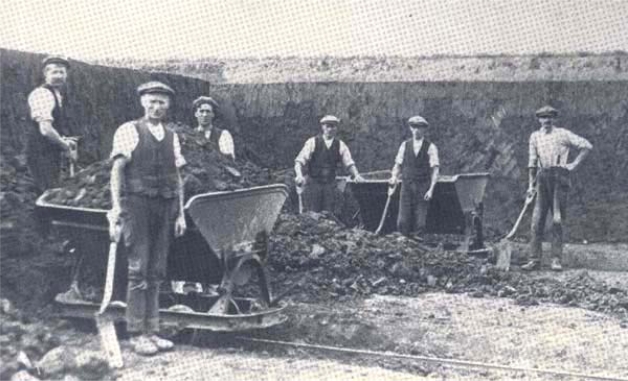
‘Moulders’ at the Murston brickfields. The ‘moulders’ shaped clay into bricks, each man making close on 1,000 every hour for an 8½ hour day and a 58 hour week. One brickie is on record as having made 986,091 bricks between April and September.

**Figure 2.**
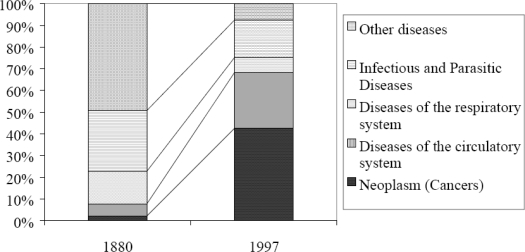
Causes of Death in England and Wales: 1880 and 1997. Reprinted from Charlton [24], vol. 2, p. 9.
